# Validation and cross-cultural adaptation of the National Eye Institute Visual Function Questionnaire (NEI VFQ-25) in Serbian patients

**DOI:** 10.1186/s12955-015-0330-5

**Published:** 2015-09-15

**Authors:** Bojan Kovac, Miroslav Vukosavljevic, Jelena Djokic Kovac, Mirko Resan, Goran Trajkovic, Janko Jankovic, Milena Smiljanic, Anita Grgurevic

**Affiliations:** Clinic for Ophthalmology, Medical Faculty of Military Medical Academy, University of Defense, Belgrade, Serbia; Clinical Center of Serbia, Faculty of Medicine, University of Belgrade, Belgrade, Serbia; Institute of Medical Statistics and Informatics, Faculty of Medicine, University of Belgrade, Belgrade, Serbia; Institute of Social Medicine, Faculty of Medicine, University of Belgrade, Belgrade, Serbia; Institute of Epidemiology, Faculty of Medicine, University of Belgrade, Visegradska 26a, PO Box 20, 11129 Belgrade 102, Serbia

## Abstract

**Purpose:**

To test the validity and reliability of the Serbian version of the interviewer-administered format of the National Eye Institute Visual Functioning Questionnaire (NEI VFQ-25).

**Methods:**

The Serbian version of NEI VFQ-25 was translated in accordance with standard methods that have been adopted internationally. In order to assess the reliability and validity of the translated NEI VFQ-25, we used a sample of 105 patients with four different chronic ocular diseases. Cronbach’s alpha coefficient was used to assess internal consistency for each subscale. To assess test–retest reliability, intraclass correlation coefficients were used. The test–retest data were obtained from clinically stable patients with age-related cataracts, in surveys performed 2 weeks apart. Rasch analysis was also applied as a modern methods of psychometric assessment of the questionnaire.

**Results:**

Four groups of patients were studied and the most prevalent were patients with cataract 40 (38.1 %), followed by diabetic retinopathy 31 (29.5 %), age related macular degeneration 22 (21.0 %) and glaucoma 12 (11.4 %). The overall index score on the NEI VFQ-25 ranged from 65.3 to 67.8 with a mean of 67.4 ± 15.0. Cronbach’s alpha coefficient (index of internal consistency reliability) ranged from 0.643 to 0.889 for the subscales. Evaluation of the validity of the Serbian version of NEI VFQ-25 is presented in the multi-trait–multi-method matrix and all items passed the convergent and discriminant validity tests. Rasch analysis showed a good measurement precision, but also demonstrated misfitting items and multidimensionality of the questionnaire.

**Conclusion:**

Although traditional validation method indicates that the Serbian version of NEI VFQ-25 is a valid and reliable instrument for the assessment of vision specific QoL in Serbian populations aged 40 years or older, Rasch analysis revealed a substantial weakness of the questionnaire that should be taken into consideration when interpreting the results.

**Electronic supplementary material:**

The online version of this article (doi:10.1186/s12955-015-0330-5) contains supplementary material, which is available to authorized users.

## Introduction

Health-related quality of life (HRQOL) is a multidimensional concept that can be described as the degree of influence of a medical condition or treatment to the usual or expected physical, emotional and social well-being. Factors that play a role in a quality of life (QoL) vary according to personal preferences. For many, however, having enough visual ability to do those things that they want to do is a high priority. Quality of vision is an integral part of HRQOL and the impact of ophthalmic diseases on QoL has been documented in a series of studies [1–3]. Patients often do not perceive the same benefit as recorded by objective measures such as visual acuity, visual field testing because the objective measurements do not evaluate the patient’s perceptions of their own disease. Numerous instruments that evaluate patients’ subjective perceptions regarding QoL have been developed so far. Although generic instruments can effectively assess HRQOL for persons with nonocular conditions, they usually cannot fully capture HRQOL in those with visual impairment [4–7]. Measuring the vision specific QoL gives us a wider view of the effect of the disease or the effect of the treatment on a patient’s life.

Many specific questionnaires for patients with visual impairment have been developed and offered to the ophthalmologists over the past twenty years [8, 9]. National Eye Institute visual function questionnaire NEI VFQ-25 was originally developed by the National Eye Institute mainly for the English-speaking North American populations [10]. It is shorter version of previously developed 51-item version [11]. The NEI VFQ-25 is a questionnaire that assesses eleven dimensions of visual function and has been proposed as a means to assess the efficacy of treatment for different ocular conditions [12]. The NEI VFQ-25 was developed in the USA and has been translated into a number of different languages: Italian, French, German, Spanish, Turkish, Chinese, Japanese, Greece, Portuguese [13–20]. To our knowledge, none of the vision-targeted health status questionnaires have been translated into Serbian, and neither has been developed in Serbian. Therefore, we decided to translate the NEI VFQ-25 questionnaire into Serbian and to assess its psychometric characteristics.

## Methods

The NEI VFQ-25 has 25 items that measure vision-targeted HRQOL and are grouped into 12 subscales: general health (GH, one item); general vision (GV, one item); ocular pain (OP, two items); difficulty with near-vision activities (NV, three items); difficulty with distance-vision activities (DV, three items); limitation of social functioning because of vision (SF, two items); mental health problems because of vision (MH, four items); role limitations because of vision (RL, two items); dependency on others because of vision (DP, three items); driving difficulties (DR, two items); difficulty with color vision (CV, one item); and difficulty with peripheral vision (PV, one item). Each subscale score is converted to a score between 0 and 100, and higher score indicates better vision-specific HRQOL. The composite NEI VFQ-25 score is the mean score of all items except for the general health item. There are 12 optional items, presented in Appendix one of the questionnaire. An investigator may select to add these items to a specific subscale if the subscale represents the main dimension of vision-targeted HRQOL that is felt to be most important for the condition under study.

### Development of the Serbian version

The Serbian version of NEI VFQ-25 was translated in accordance with standard methods that have been adopted internationally [21], including forward translation, backtranslation, examination of the translation quality and adjudication by bilingual speakers, and a pilot test on ten patients who visited the outpatient service of our clinic for a check-up. The results of the pilot-testing indicated that the instrument was well accepted, as it was short in duration (about 10 min) and all items were easy to understand. Pilot testing was used as cognitive debriefing and adaptation of the questionnaire to the experience of Serbian patients mandated slight modification of only two questions. Thus, item ‘13’ (How much difficulty do you have visiting people at their homes, at parties, or in restaurants?) was translated as: (How much difficulty do you have visiting people at their homes, gatherings or restaurants?). Due to low popularity of golf in Serbia, golf has been changed into riding bicycle in item A7. This study was performed in accordance with the Declaration of Helsinki. The Ethical Committee of the Faculty of Medicine, University of Belgrade reviewed and approved the study. All participants provided signed informed consent before enrolment.

### Study design and population

The study was conducted between December 2013 and July 2014 at Eye clinic of Military Medical Academy, Belgrade and 105 patients were included. In order to assess the reliability and validity of the translated NEI VFQ-25, we used a sample of four patient groups: patients with cataract (C), age-related macular degeneration (ARMD), glaucoma (G) and diabetic retinopathy (DR). All surveys were administered by two trained physicians using a face*-*to*-*face interview method. The following instruments were used: the Serbian version of the NEI VFQ-25, the questionnaire with 12 optional items related to different aspects of vision-specific HRQOL, and the SF-36 health survey questionnaire. The SF-36 was chosen because it is one of the most widely used measures in health services research and has been already translated into the Serbian language and validated [22]. This questionnaire includes 8 subscales: general health, physical function, physical role activities, usual emotional role activities, mental health, social function, vitality, and bodily pain. Each of the subscales is scored on a 0 to 100 scale, in which 100 indicates the best possible score and zero indicates the worst function.

Eligibility criteria included an age of 40 years and older, presenting visual acuity (VA) of 0.6 or worse in the better eye, Serbian speaking, no cognitive or hearing impairment, no motion impairment, and no history of laser or incisional eye surgery within 3 months. All patients underwent a complete ophthalmologic examination, including best corrected VA testing, slitlamp biomicroscopy, dilated fundus examination, and Goldmann applanation tonometry. All glaucoma patients exhibited glaucomatous disc cupping and visual field examination utilized the G2 program, Octopus 101 Perimeter System (HAAG-STREIT AG, Koeniz-Berne, Switzerland). Glaucoma patients with any ocular pathology other than mild nuclear sclerosis were excluded. Patients with age related macular degeneration (ARMD) had at least one of the following features consistent with ARMD, namely, geographical atrophy in the macula, a pigment epithelial detachment or choroidal neovascularization. Patients with late sequelae of ARMD, such as scarring in the macula, were included in the study, and pseudophakia was not considered as an exclusion criterion for ARMD patients. The pattern of cataract was noted as nuclear, subcapsular, or cortical. The severity of age-related cataracts was graded with the Lens Opacities Classification System III (slit lamp, standard testing conditions) [23]. Cataract patients with any other ocular pathology were excluded. Grading protocols for DR were modifications of the Early Treatment Diabetic Retinopathy Study adaptation of the modified Airlie House classification of DR [24]. Diabetic retinopathy was classified as 1: nonproliferative DR (NPDR), mild, moderate, or severe; or 2: proliferative (PDR). Fundus fluorescein angiography was performed in diabetic patients who had macular involvement.

### Statistical analysis

The statistical analysis consisted of reliability and validity analyses which were done with SPSS version 21.0 for Windows (SPSS Inc. Chicago, IL).

### Descriptive analysis and item analysis

The item analysis was performed using the data from the different subject groups. The percentage of missing values was examined for each item. We also examined whether each item’s distribution of responses was strongly skewed (large ceiling effect or floor effect).

### Reliability

Cross-sectional data from the four patient groups were used to quantify reliability. Cronbach’s alpha coefficient was used to assess internal consistency for each subscale [25]. The item-total score correlations were explored by Spearman’s correlation analysis. According to the general guidelines suggested by Colton, correlations ranging from 0.00 to 0.25 indicate little or no relationship; those from 0.25 to 0.50 suggest a fair degree of relationship; values of 0.50–0.75 are moderate to good; and values above 0.75 are considered good to excellent [26]. To assess test–retest reliability, intraclass correlation coefficients were used. The test–retest data were obtained from clinically stable patients with age-related cataracts, in surveys performed 2 weeks apart. The time interval was recommended by Streiner and Norman [27, 28].

### Validity

Multi-trait analysis was used to evaluate convergent and discriminant validity according to Campbell ad Fiske [29]. Each item was hypothesized to belong to only one multi-item subscale and correlations between the score on that item and the scores on all the subscales were computed. For each item, if the correlation between the score on that item and the score on the subscale to which that item belongs is 0.4 or higher, that item is said to have ‘passed’ the test of convergent validity. On the other hand, for each item, if the correlation between the score on that item and the score on the subscale to which that item belongs is greater than the correlations between the score on that item and the scores on all the subscales to which that item does not belong, then that item is said to have ‘passed’ the test of discriminant validity. To assess concurrent validity, correlations between scores on the NEI VFQ-25 and scores on the SF-36 subscales were computed. We hypothesized that the NEI VFQ-25 ‘Mental health’, ‘Social functioning’ and ‘Dependency’ scores would be associated more strongly with the SF-36 subscale scores that measured similar domains. The clinical validity was examined by correlation of clinical measurements (visual acuity (VA) and visual field deficit) and scores of all subscales. We computed the correlations between subscale scores and VA with best correction in the better and worse eye and deficits in visual fields as measured by the Octopus perimeter in the better and worse eye. Finally, we used factor analysis to assess the uni-dimensionality of the scale, in preparation for computing a composite score. Factor analysis was done using 11 subscales (‘Driving’ was not included), with the maximum-likelihood solution and varimax rotation. The ‘Driving’ subscale was not included because 73.3 % of the responses on this subscale were missing.

### Rasch analysis

Alongside the traditional methods, the psychometric properties of the Serbian NEI VFQ-25 were also evaluated by Rasch analysis. The purposes of Rasch analysis are to maximize the homogeneity of the trait and to allow greater reduction of redundancy at no sacrifice of measurement information by decreasing items and/or scoring levels to yield a more valid and simple measure. Rasch analysis consists of the following components: category threshold order, person separation, unidimensionality, targeting, and differential item functioning (DIF). Winsteps (version 3.90) was used to perform Rasch analysis using the Andrich rating scale model [30]. Numerical responses for each item were recoded so that one was assigned as the lowest possible response and five as the highest. The ranking of response categories was reversed when necessary so that higher scores always represented higher levels of visual functioning.

#### Category Threshold Order

The first step was to examine the ordering of the response category threshold. Disordering of categories occurs when categories are underused, have unclear definition, or when the number of categories exceed the number of levels that participants can distinguish. Disordered thresholds can be a cause of item misfit. Therefore, in a case of disordered thresholds, combining adjacent categories was done until thresholds were ordered; this was made before further analyses were carried out.

#### Person separation

Person separation is a measure of questionnaire’s precision and can be used to estimate how many groups or strata of person ability can be discriminated. A person separation reliability of 0.8 was the minimum value of discrimination for an instrument in this study; it means that three strata can be discriminated, and a reliability coefficient of 0.9 indicates four strata. The person separation index is the ratio of the variance in the person measures for the sample to the average error in estimating these measures. A person separation index of ≥2.0 represents the minimum acceptable level of separation.

#### Unidimensionality

Unidimensionality refers to whether the questionnaire measures a single underlying construct. Dimensionality is assessed by using item-fit statistics (mean square statistics) and by principal component analysis (PCA) of the residuals (difference between the observed and expected responses). There are two types of fit statistics, infit and outfit. Infit statistic is more sensitive to the pattern of responses to person-targeted items and less sensitive to the presence of outliers and therefore is considered more informative. Instrument was evaluated using the parameters proposed by Pesudovs et al. [6–31]. Fit statistics between 0.7 and 1.3 are considered acceptable [30] though a more yielding criterion of between 0.5 and 1.5 is also considered useful for the measurement [32]. Data are considered unidimensional if most of variance is explained by the principal component (>60 %) and if there is no significant explanation of the residual variance by the contrasts to the principal component. The unexplained variance by the contrast should be less than two eigenvalue units.

#### Targeting

Targeting refers to how well the difficulty of items in the scale matches the abilities of the persons in the sample. It can be evaluated by visually inspecting person-item maps and by measuring the difference between person and item mean values. A difference between means of more than 1 logit points out notable mistargeting.

#### Differential Item Functioning (DIF)

DIF was carried out to assess whether the items function similarly for persons at the same level of ability regardless of their characteristics. For DIF testing, the respondents were stratified by sex, age (≤70 years and >70), systemic comorbidity (present/absent) and better eye visual acuity (≤0.4 and > 0.4). DIF was considered absent if a difference was less than 0.5 logits, minimal if it ranged from 0.5 to 1.0 logits and notable if it was greater than 1.0 logit [33]. The 12 subscales were analyzed separately using the same procedures and criteria for reliability and validity that were used for the overall questionnaire. However four subscales (general health, general vision, color vision, and peripheral vision) contain only one item each and do not fulfill the criteria to perform Rasch analysis. The person separation reliability was used to evaluate the appropriateness of use of the subscales.

## Results

The mean age of the patients included in the study was 69.2 ± 9.9 years (mean ± SD). Among those patients 42 (40 %) were males, and 63 (60 %) were females. Four groups of patients were studied and the most prevalent were patients with cataract 40 (38.1 %), followed by DR 31 (29.5 %), ARMD 22 (21.0 %) and glaucoma 12 (11.4 %). Demographics and clinical data, including marital, educational, working status, visual acuity and comorbidity for the participants are presented in Table 1. The subscale and composite scores of patients with different eye diseases are given in Table 2. The overall index score on the NEI VFQ-25 ranged from 65.3 to 67.8 with a mean of 67.4 ± 15.0. The highest missing values were identified in the questions regarding ‘Driving’ (missing percentages of 59.0 % and 73.3 % in items 15 and 16, respectively). Ceiling and floor values of the sample suggested that the data were moderately skewed (Additional file 1).

**Table 1:** Demographic and clinical characteristic of the study sample

Variables	Sample, *n* = 105
*Age*, mean + SD	
Overall	69.22 ± 9.89
Cataract patients	69.62 ± 10.66
Glaucoma patients	70.00 ± 8.60
ARMD patients	74.18 ± 6.42
DR patients	64.90 ± 9.89
*Gender*, number (%)	
Male	42 (40.0)
Female	63 (60.0)
*Marital status*, number (%)	
Married	70 (66.7)
Non-married	5 (4.8)
Widowed	26 (4.8)
Divorced	4 (8.0)
*Educational status*, number (%)	
Elementary school (1-8 years)	7 (6.7)
Secondary school	53 (50.5)
Higher school	16 (15.2)
University degree	29 (27.6)
*Working status*, number (%)	
Working	16 (15.2)
Not working/pensioner	89 (84.8)
*Ophthalmic disease*, number (%)	
Cataract	40 (38.1)
Glaucoma	12 (11.4)
ARMD	22 (21.0)
DR	31 (29.5)
*Visual acuity (Snellen)*, mean (range)	
Better eye	0.42 (0.01-0.06)
Worse eye	0.20 (0.01-0.06)
*Comorbidity (systemic)*, number (%)	
Non	16 (15.2)
One	59 (56.2)
Two	22 (21.0)
Three or more	8 (7.6)

**Table 2:** Subscale and overallscores (mean ± SD) of study subjects according to the type of diagnosis

	Cataract	DR	ARMD	Glaucoma	Total
General health	49.3 ± 13.7	33.8 ± 26.2	35.2 ± 28.5	39.5 ± 16.7	40.7 ± 27.5
General vision	54.0 ± 13.7	57.4 ± 18.4	48.1 ± 20.1	61.6 ± 13.3	54.6 ± 16.9
Ocular pain	87.8 ± 15.8	90.7 ± 13.2	84.0 ± 19.7	62.5 ± 20.6	85.0 ± 18.4
Near activities	48.9 ± 21.6	54.7 ± 28.4	49.2 ± 19.9	69.4 ± 20.7	53.0 ± 23.9
Distance activities	58.5 ± 20.8	63.1 ± 27.0	60.6 ± 29.1	69.4 ± 24.9	61.5 ± 24.9
Social functioning	85.0 ± 21.7	83.0 ± 18.9	78.9 ± 28.9	85.4 ± 19.8	83.2 ± 22.3
Mental health	68.9 ± 21.3	67.0 ± 20.8	67.3 ± 18.6	47.9 ± 25.4	65.6 ± 21.8
Role difficulties	68.5 ± 26.8	59.6 ± 28.8	59.6 ± 20.7	58.3 ± 25.1	62.9 ± 26.1
Dependency	58.7 ± 15.8	53.7 ± 15.9	56.9 ± 12.4	52.4 ± 11.9	56.1 ± 14.8
Driving	9.54 ± 42.2	53.9 ± 38.5	47.2 ± 43.1	66.6 ± 14.4	47.3 ± 39.8
Color vision	95.0 ± 11.6	93.5 ± 27.1	94.3 ± 17.1	85.4 ± 16.7	93.3 ± 14.8
Peripheral vision	60.6 ± 25.2	69.3 ± 27.1	85.2 ± 26.3	62.5 ± 19.9	68.5 ± 26.8
Composite score	67.7 ± 13.2	67.8 ± 17.3	67.4 ± 15.4	65.3 ± 15.5	67.4 ± 15.0

*DR* - diabetic retinopathy

*ARMD* - age-related macular degeneration

### Reliability

Evaluation of the reliability of the Serbian version of the NEI VFQ-25 is presented in Table 3. Cronbach’s alpha coefficient (index of internal consistency reliability) ranged from 0.643 to 0.889 for the subscales. The majority of the subscales presented high internal consistency. We had one subscale with Cronbach’s alpha below 0.7. The lowest Cronbach’s alpha value was obtained for the ‘Vision specific social functioning’ (VSSF, 0.643). VSSF subscale had Cronbach’s alpha higher than 0.7 in testing with optional items (VSSF, 0.724). The highest Cronbach alpha values were obtained for ‘Driving’ (D, 0.889), followed by ‘Near activities’ (NA, 0.827), ‘Role difficulties’ (RD, 0.804), ‘Distance activities’ (DA, 0.785), ‘Ocular pain’ (OP, 0.746) and ‘Mental health’ (MH, 0.719). Regarding test-retest reliability, the intraclass correlation coefficient was higher than 0.7 for all of the subscales. The highest value was obtained for the ‘General health’ (0.986).

**Table 3:** Reliability and validity analysis

Subscale	Number of items	Interclass correlation coefficients (95 % CI)	Cronbach’s alpha (95 % CI)	Range of item-scale correlations	Convergent validity	Discriminant validity
General health	1	0.986 (0.947-0.996)	NA	NA	NA	NA
General vision	1	0.808 (0.285-0.948)	NA	NA	NA	NA
Ocular pain	2	0.941 (0.780-0.984)	0.746 (0.626-0.827)	0.886-0.900	100	100
Near activities	3	0.958 (0.844-0.989)	0.827 (0.760-0.878)	0.816-0.897	100	100
Distance activities	3	0.947 (0.803-0.986)	0.785 (0.679-0.860)	0.799-0.850	100	100
Social functioning	2	0.968 (0.880-0.991)	0.643 (0.467-0.761)	0.870-0.877	100	100
Mental health	4	0.904 (0.645-0.974)	0.728 (0.630-0.804)	0.599-0.872	100	100
Role difficulties	2	0.965 (0.871-0.991)	0.804 (0.712-0.867)	0.904-0.927	100	100
Dependency	3	0.895 (0.610-0.972)	0.824 (0.756-0.875)	0.740-0.907	100	100
Driving	3	NA	0.889 (0.787-0.947)	0.898-0.964	100	100
Color vision	1	0.933 (0.752-0.982)	NA	NA	NA	NA
Peripheral vision	1	0.964 (0.865-0.990)	NA	NA	NA	NA

*NA*- not applicable (needs two or more items), *CI* confidence interval

### Validity

Evaluation of the validity of the Serbian version of NEI VFQ-25 is presented in the multi-trait–multi-method matrix (Table 3). All items passed the convergent and discriminant validity tests. For concurrent validity, strong Spearman correlations were detected between scores on most of the NEI VFQ-25 subscales and similar domains of the SF-36 (Table 4). ‘Dependency’ and ‘Mental health’ in NEI VFQ-25 highly correlated with all subscales in SF-36. ‘Role emotional’ correlated only with ‘Ocular pain’, ‘Mental health’ and ‘Color vision’. There were no correlations between ‘Driving’ and all of the SF-36 subscales. The impact of visual acuity and visual field deficits on vision-specific quality of life is presented in Table 5. The ‘General health’ and ‘Ocular pain’ subscales poorly correlated with visual acuity of the better eye. All the other subscales highly correlated with better eye visual acuity. ‘General health’, ‘Ocular pain’, ‘Mental health’, ‘Driving’ and ‘Color vision’ poorly correlated with visual acuity of the worse eye. Strong correlations were detected between best corrected visual acuity (BCVA) and all subscales except for ‘General health’ and ‘Ocular pain’. Particularly strong correlation was detected between BCVA and subscales that are associated with central vision (*i.e.* ‘Near activities’ and ‘Distance activities’). The results of factor analysis (FA) performed with ten subscales (‘General Health’ and ‘Driving’ were excluded) are shown in Table 6. Two factors were obtained. The ‘General vision’, ‘Near activities’, ‘Distance activities’, ‘Social function’, ‘Role difficulty’, ‘Peripheral vision’ subscales were included in factor one. The ‘Mental health’, ‘Ocular pain’, ‘Dependency’ and ‘Color vision’ subscales were included in the second factor.

**Table 4:** Correlation of NEI VFQ-25 subscales with subscales of the SF-36

SF-36								
NEI VFQ-25	Physical functioning	Role physical	Role emotional	Energy fatigue	Emotional wellbeing	Social functioning	Pain	General health
General health	0.434**	0.308**	0.057	0.512**	**−**0.342**	0.420**	0.251**	0.716**
General vision	0.486**	0.393**	−0.078	0.299**	−0.266**	0.255**	0.373**	0.402**
Ocular pain	0.375**	0.417**	0.330**	0.188	−0.445**	0.429**	0.223*	0.283**
Near activities	0.322**	0.249**	−0.093	0.333**	−0.134	0.147	0.320**	0.242*
Distance activities	0.403**	0.376**	−0.007	0.371**	−0.322**	0.324**	0.360**	0.313**
Social function	0.457**	0.398**	0.073	0.351**	−0.109	0.277**	0.458**	0.257**
Mental health	0.532**	0.432**	0.229*	0.391**	−0.409**	0.420**	0.915**	0.336**
Roles difficulties	0.513**	0.468**	0.034	0.479**	−0.319**	0.484**	0.515**	0.319**
Dependency	0.568**	0.434**	0.147	0.519**	−0.490**	0.527**	0.725**	0.339**
Driving	−0.178	−0.053	0.137	0.062	−0.215	0.238	0.155	−0.111
Color vision	0.313**	0.289**	0.193*	0.257**	−0.213*	0.357**	0.299**	0.016
Peripheral vision	0.275**	0.295**	0.180	0.147	−0.116	0.148	0.205*	0.085

*Significant at the 0.05 level (2-tailed)

**Significant at the 0.01 level (2-tailed)

**Table 5:** Spearman’s correlations of NEI VFQ 25 subscales with visual acuity and visual field

NEI VFQ 25 subscales	BCVA better eye correlation (*p*-value)	BCVA worse eye correlation (*p*-value)	MD better eye correlation (*p*-value)	MD worse eye correlation (*p*-value)
General health	0.158 (0.107)	0.145 (0.139)	−0.340 (0.279)	−0.235 (0.463)
General vision	0.432** (<0.001)	0.329** (0.001)	−0.293 (0.356)	−0.454 (0.138)
Ocular pain	0.100 (0.313)	0.104 (0.290)	−0.674* (0.016)	−0.395 (0.203)
Near activities	0.578** (<0.001)	0.367** (<0.001)	−0.226 (0.480)	−0.241 (0.451)
Distant activities	0.712** (<0.001)	0.476** (<0.001)	−0.267 (0.402)	**−**0.547 (0.066)
Social functioning	0.514** (<0.001)	0.275* (0.005)	−0.543 (0.068)	−0.364 (0.245)
Mental health	0.250* (0.010)	0.202* (0.039)	−0.430 (0.163)	−0.200 (0.532)
Role difficulties	0.535** (<0.001)	0.267* (0.006)	−0.515 (0.087)	**−**0.498 **(**0.099)
Dependency	0.328** (<0.001)	0.238* (0.014)	*−*0.472 (0.122)	−0.427 (0.167)
Driving	0.502** (0.001)	0.260 (0.088)	1.000** (<0.001)	−0.500 (0.667)
Color vision	0.319** (0.001)	0.078 (0.429)	−0.865** (<0.001)	**−** 0.847** (0.001)
Peripheral vision	0.370** (<0.001)	0.388** (<0.001)	−0.342 (0.276)	−0.555 (0.061)
Composite score	0.598** (<0.001)	0.384** (<0.001)	0.529 (0.077)	0.535 (0.073)

*Significant at the 0.05 level (2-tailed)

**Significant at the 0.01 level (2-tailed)

Italic characters indicate statistical significant correlation coefficient of 0.4 or greater

*MD* mean defect (only glaucoma patients)

**Table 6:** Results of factor analysis on ten subscales of NEI VFQ-25 (‘General Health’ and ‘Driving’ were excluded): factor loadings after varimax rotation

Subscale	Factor 1	Factor 2
Near activities	*0.882*	0.082
Distance activities	*0.879*	0.199
Social functioning	*0.763*	0.404
General vision	*0.757*	0.128
Role difficulties	*0.655*	0.363
Peripheral vision	*0.622*	0.263
Mental health	0.256	*0.816*
Dependency	0.364	*0.732*
Ocular pain	−0.005	*0.689*
Color vision	0.389	*0.541*

### Rasch analysis

#### Response category assessment

The Rasch model showed disordered thresholds for six items which belong to one of the two rating scales (Difficulty Scale and Agreement Scale). There was an overlap between categories one and two for the items that belong to the Difficulty Scale and combining these categories repaired disorder. For the items that belong to the Agreement rating scale with response options ranging from definitely true to definitely false there was an overlap between categories two and three. Because category three („not sure“) is a neutral category and only a small percent of the participants chose this option it was coded as a missing category, and therefore category thresholds were ordered properly.

#### Item Fit statistics

On the NEI VFQ-25, five items showed misfit with infit mean scores > 1.3, suggesting that the items introduced noise into the data and did not measure the underlying construct. These items belonged to the ‘Driving’ subscale with a high percent of missing data (73.3 %), ‘Distance activities’ (Going out to movies/plays/sports events), ‘Mental health’ subscale (Embarrassment) and ‘General health’. Removal of these items improved the fit of the scale to the Rasch model. Fit statistics of the remaining items are presented in Table 7.

**Table 7:** Fit statistics after removal of misfitting items

Items	Measure	Error	Infit MNSQ	Outfit MNSQ
Reading normal newsprint	1.54	0.11	0.62	0.63
Seeing well up close	1.13	0.11	0.87	0.97
Less control	0.95	0.11	1.09	1.16
Worry about eyesight	0.77	0.11	1.26	1.39
Going downstairs at night	0.64	0.11	0.81	0.87
Accomplish less	0.64	0.11	0.80	0.79
Reading street signs	0.44	0.11	0.99	0.94
Limited in endurance	0.38	0.11	1.23	1.29
Seeing objects off to side	0.34	0.11	1.39	1.31
General vision	0.15	0.11	0.55	0.51
Finding something on crowded shelf	0.14	0.11	0.90	0.92
Frustrated	0.00	0.12	1.26	1.23
Stay home most of the time	−0.12	0.12	1.30	1.09
Rely too much on others’ words	−0.68	0.13	0.99	0.90
Visiting others	−0.79	0.14	0.87	0.67
Seeing how people react	−0.84	0.14	1.03	0.89
Amount of pain or discomfort	−0.88	0.14	1.27	1.23
Amount of time in pain	−0.94	0.14	1.33	1.52
Need much help from others	−0.96	0.14	0.68	0.61
Matching clothes	−1.90	0.20	0.89	0.64

#### Person separation

**Fig. 1:**
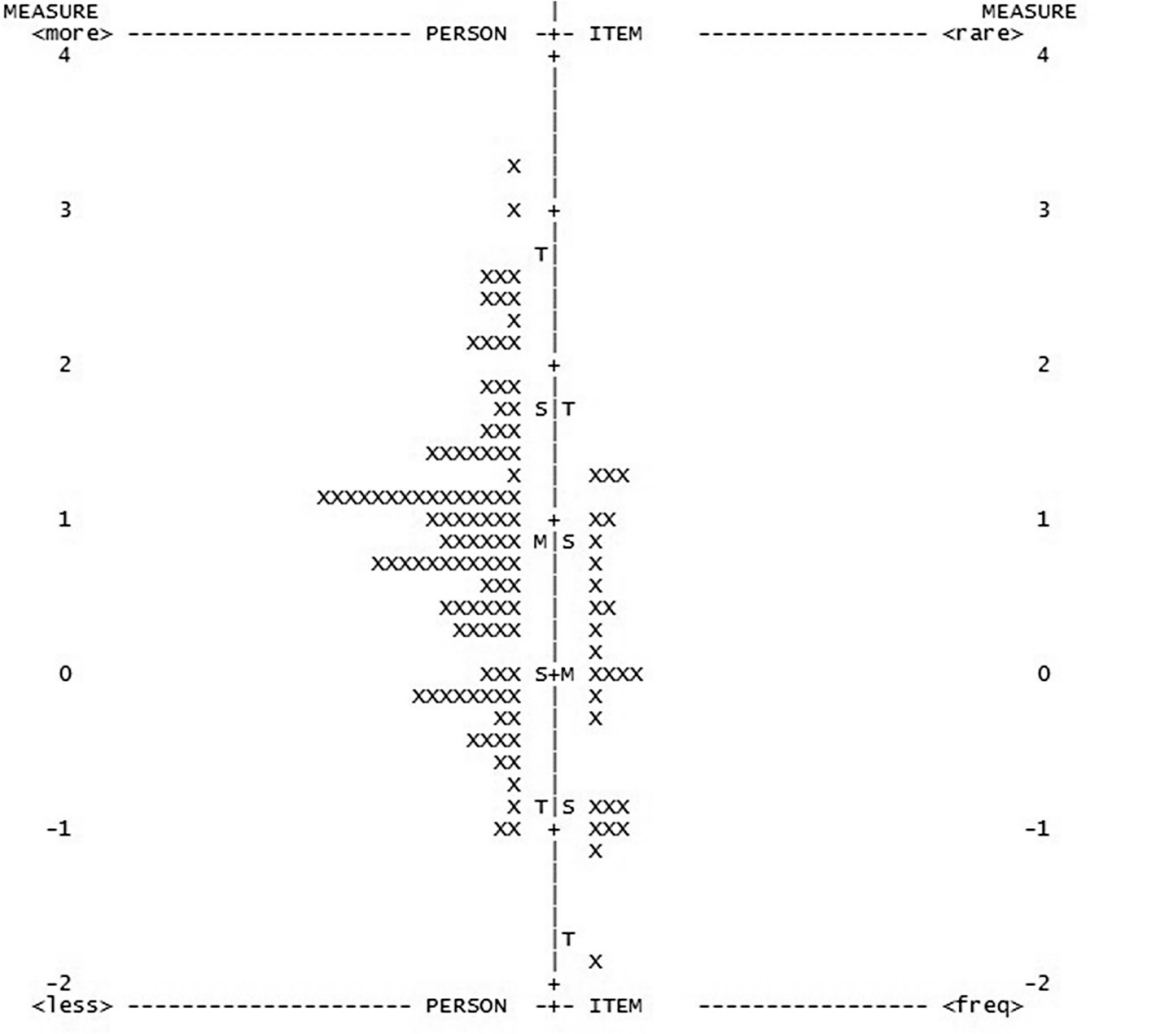
Person-item map of the NEI VFQ-25. The participants are on the left of the dashed line, with more able participants located at the top of the map. Items are located on the right of the dashed line, with more difficult items located at the top of the map. (M = mean; S = 1 SD from the mean; T = 2 SD from the mean)

**Fig. 2:**
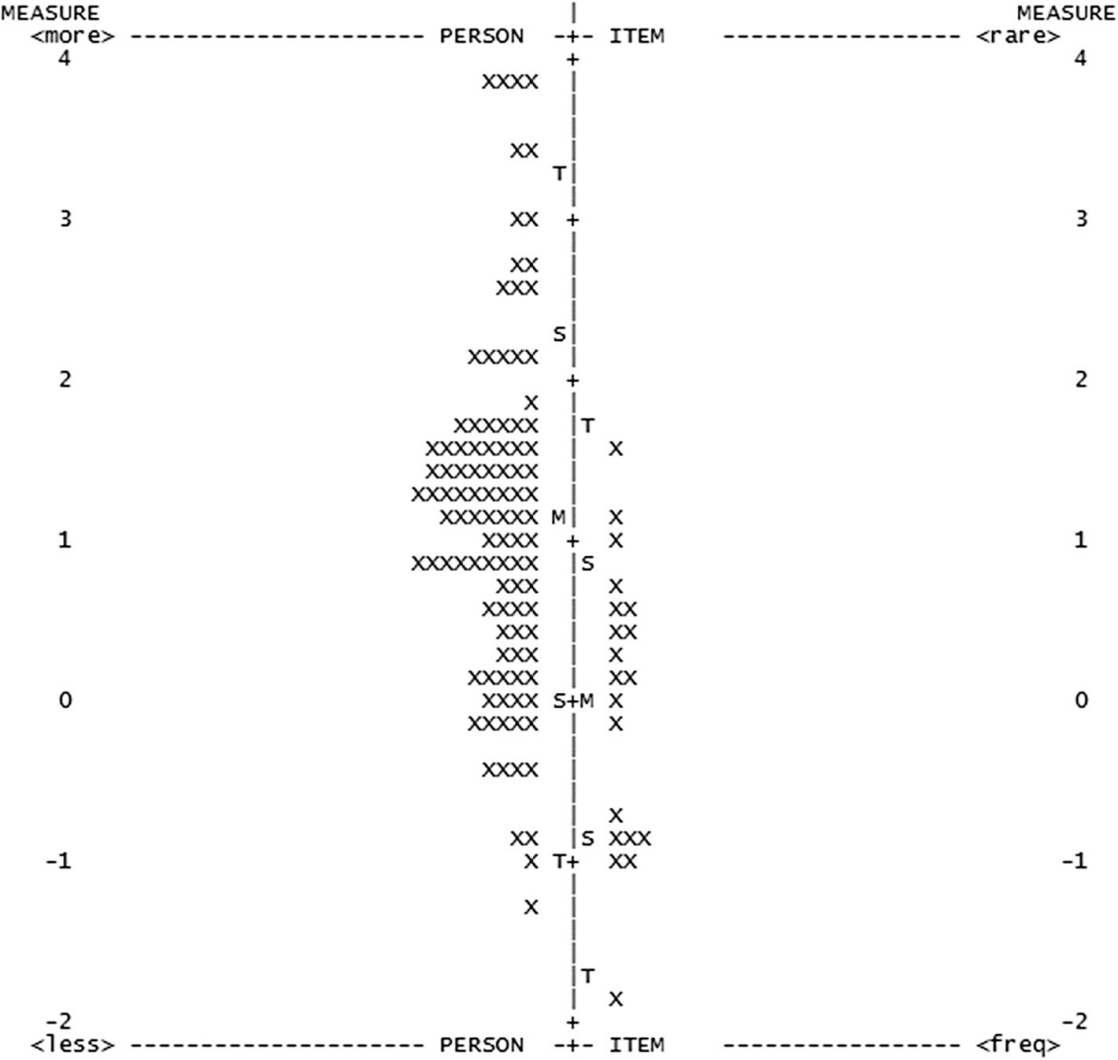
Person-item map of the NEI VFQ-25 after removal of misfitting items. The participants are on the left of the dashed line, with more able participants located at the top of the map. Items are located on the right of the dashed line, with more difficult items located at the top of the map. (M = mean; S = 1 SD from the mean; T = 2 SD from the mean)

Person separation reliability coefficient was 0.91 indicating excellent discrimination of the instrument between the persons of different abilities. The person separation index was 3.26 (Table 8). Targeting was examined by person item maps. Items were not ideally matched to persons in the sample for original version NEI VFQ-25 (Fig. 1) and neither were for version after removal of misfitting items (Fig. 2). Most of the items cover people with low and moderate visual ability and most of uncovered percentage represents persons with high visual ability.

**Table 8:** Overall performance of the NEI VFQ-25

Parametar	NEI VFQ-25
Misfitting items (n)	5
Person separation reliability (PSR)	0.91
Person separation index (PSI)	3.26
Principal component analysis (eigenvalue in 1st contrast)	3.3
Valid subscales (n)	3
DIF by age (2 items)	≤0.61 logits
DIF by sex (3 items)	≤0.61 logits
DIF by systemic comorbidity (1 item)	0.94 logits
DIF by better eye visual acuity (5 items)	≤0.87 logits

#### Dimensionality

The PCA of the residuals showed that the variance explained by measures for the empiric calculation (54.4 %) was comparable to that explained by model (54.8 %). The first contrast in the residuals explained 8.5 % of the variance, and the eigenvalue of the unexplained variance in the first contrast was 3.3, suggesting presence of a second dimension in the scale. The unexplained variance by the second contrast was 2.3 eigenvalue units and no further contrasts exceeded 2.0 eigenvalue units. Five items loaded positively onto the first contrast (correlation > 0.4) and belonged to the ‘Role difficulties’ (three items), and ‘Mental health’ (two items) subscales. Four items loaded positively onto the second contrast and belonged to the ‘Ocular pain (two items), ‘Distance activities’ (one item), and ‘Peripheral vision’ (one item) subscales.

#### Differential item functioning

Three items showed differential item functioning by sex (Worry about eyesight (3), Stay home most of the time (13), Relay too much on others’ words (23), although it was minimal magnitude ≤ 0.61 logits. Two items demonstrated minimal differential item functioning by age: Seeing how people react (11), and Limited endurance (18), with logit values 0.61 and 0.59, respectively. Five items (3, 8, 11, 19, 22) displayed differential item functioning by better eye visual acuity with a logit values ≤ 0.87. One item (Stay home most of time) showed differential item functioning by systemic comorbidity (0.94 logits). No item showed notable DIF.

#### Subscales analysis

Rasch analysis showed that only three of eight subscales had satisfactory performance in person separation reliability; ‘Driving’ 0.9, ‘Near activities’ 0.86 and Role Difficulties 0.85.

#### Reengineering the NEI VFQ-25

Following the examples of Pesudovs et al. [31], Mollazadegen et al. [34] and Marella et al. [33] two separate scales were formed: the visual functioning scale and the socioemotional scale. New scales were developed by removing the most misfitting items in an iterative process. Among items which loaded onto the visual functioning construct nine items fit the Rasch model (Table 9). The PCA of the residuals showed that the variance explained by measures for the empiric calculation (71.0 %) was comparable to that explained by model (70.8 %). The eigenvalue of the unexplained variance in the first contrast was 1.77 units. There were no misfitting items. Person separation reliability coefficient was 0.89. The targeting was slightly worse than that of original version and was suboptimum (1.43 logits). There was no DIF. Among items which loaded onto the socioemotional construct, nine items fit the Rasch model (Table 9). The PCA of the residuals showed that the variance explained by measures for the empiric calculation (59.2 %) was comparable to that explained by model (59.6 %). The eigenvalue of the unexplained variance in the first contrast was 1.76 units. Two items slightly misfit the model at level >1.3 (1.34; 1.38). Person separation reliability coefficient was 0.83. The targeting was slightly worse than that of original version and was suboptimum (1.48 logits). There was no DIF.

**Table 9:** Rasch analysis fit statistics of the two-factor model scales

Scales	Items in scale	Misfitting items	Person separation reliability	Person separation index	Mean person mesure (logits)	Principal component analysis (eigenvalue 1st contrast)
Visul functioning	9	0	0.89	2.86	1.43	1.77
Socioemotional	9	2^a^	0.83	2.24	1.48	1.76

^a^infit > 1.3 (1.34, 1.38)

## Discussion

Traditional clinical measures of vision may fail to assess many aspects of visual disability that are identified by individuals as being important for their daily functioning and well being [2, 4]. Many specific questionnaires for patients with visual impairment have been developed and offered to the ophthalmologists over the past twenty years [8]. To this date no questionnaires measuring vision related QoL have been developed in Serbian, and none of the vision-related QoL questionnaires have been translated and validated into Serbian. Keeping in mind the growing interest among medical professionals in Serbia for vision related QoL we decided to translate and validate the NEI VFQ-25 into Serbian.

The primary objective of our study was the evaluation of the reliability and validity of the NEI VFQ-25 in native Serbian populations with a series of most common ophthalmic diseases. Proper adaptation of the instrument to the Serbian population required a slight modification of some items. Due to suggestion proposed during the cognitive debriefing sessions item 13 “visiting with people in their homes, at parties, or in restaurants” has been changed into “visiting with people in their homes, at gatherings, or in restaurants”. In item A7 that includes sports, playing golf was changed to riding a bicycle. Minor modifications of some items during the translation and validation of the NEI VFQ-25 were also considered necessary in other populations [16–19]. Similar to the original validation studies in other populations, relatively high missing rates were encountered in the ‘Driving’ subscales. In our study relatively high missing rate (32.4 %) was found in item 14 related to ‘Distance vision’ (going out to see movies, plays, or sports events). One of possible explanation could be connected with poor economic situation in our country. However, the missing rates of the other items were comparably lower than the ones encountered during the translation and validation of the same instrument in other populations [16–19]. High ceiling percentages were encountered in some items (*i.e.* ‘Color vision: difficulty matching clothes’, ‘Mental health: Amount true: embarrassment’) and moderate skewing of data was detected. The reliability of the Serbian version of the NEI VFQ-25 was tested by internal consistency (IC) and item-scale correlations. Cronbach alpha values as measure of the IC of the scale, were satisfactory in almost all of the subscales and the overall index. The lowest value of Cronbach’s alpha was detected in ‘Social functioning’ (0.643) subscales. After inclusion of optional items for this subscale, Cronbach’s alpha value was higher than 0.7. The subscales of the Serbian version of NEI VFQ-25 presented variable but adequate internal consistencies indicating high reliability of the instrument in the population studied. The high test-retest reproducibility of the NEI VFQ-25 is a critical characteristic for a questionnaire to be used in follow-up studies. A correlation coefficient greater than 0.80 for two administrations of a scale one to two weeks apart suggests adequate stability [30]. The test-retest reliability ranged from 0.808 to 0.986 in our study. All subscales had intraclass correlation coefficient abowe 0.8. Good test–retest reliability was indicated by the high values of the intraclass correlation coefficients. Regarding the construct validation of the questionnaire, none of the items failed either the convergent or the discriminant tests. Similar findings were observed in other studies [17, 19]. The ability of the questionnaire to demonstrate the problem of different levels of VA loss also indicated a satisfactory clinical validity. Strong correlations were detected between BCVA of the subjects and the all subscales except ‘General health’ and ‘Ocular pain’. Similar correlations between VA and NEI VFQ-25 subscales have been detected by previous investigators during the validation of the instrument in other languages as well [16–20]. We also tested the validity of our version by comparison of its subscales with scales of similar content of the SF-36. The ‘General vision’ subscale showed high correlation with physical component of SF-36. The ‘Mental health’ and ‘Dependency’ subscales showed high correlation with almost all subscales of SF-36. Other NEI VFQ-25 subscales were moderately correlated with similar SF-36 subscales, except ‘Driving’ which was not correlated with any of them. It could be because of high rate of missing responses in ‘Driving’ subscale.

Factor analysis indicated that the most of the subscales that are influenced by central vision and peripheral vision correlated with the first factor, while the ‘Color vision’, ‘Ocular pain’ ‘Social functioning’ and ‘Dependency’ subscales were included in the second factor. These results are consistent with the results of previous studies, that most of subscales of NEI VFQ-25 belong to the same underlying dimension, especially connected with central vision [18, 20].

Besides traditional methods, Rasch analysis was also applied to assess psychometrics properties of NEI VFQ-25. Rasch analysis focuses on analysis at a person and item level versus test level. As opposed to traditional psychometrics, Rasch provides detailed information on rating scales, items, persons, and other factors such as rater severity [35]. Rasch analysis revealed a substantial weakness of the questionnaire that should be taken into consideration when interpreting the results.

Items belonging to the ‘General health’, ‘Driving’ subscale, ‘Distance activities’ (Going out to movies/plays/sports events), and ‘Mental health’ subscale (Embarrassment) did not fit the overall scale. Similar results were reported by other authors [19, 31, 33,]. A high percentage of missing values for subscale ‘Driving’ was also found in different population [31, 33, 34]. The categories for two rating scales (Difficulty Scale and Agreement Scale) had to be collapsed to a four-category response scale (6 items), which is in agreement with some previous studies [36, 37]. There are also studies in which categories had to be collapsed to a dichotomous scale [33]. Rasch analysis in our study reveals multidimensionality of the NEI VFQ-25 questionnaire. This result is consistent with findings in earlier studies [31, 33, 34]. The problem with multidimensionality is that the use of composite score requires that only a single construct is being measured. The results of our principal component analysis indicated that five items loaded positively onto the first contrast and belonged to the ‘Role difficulties’ (three items), and ‘Mental health’ (two items) subscales. Similar results were found in study published by Marella et al. [33] and study of Pesudovs at al. [31] in which several of the items loaded positively onto the first contrast and belonged to the ‘Role difficulties’, ‘Mental health’ and ‘Dependency’ subscales. Examination of targeting showed that most of items cover people with low and moderate visual ability and most of uncovered percentage represents persons with high visual ability. However, this finding indicates that this instrument is suitable for medical application where it should measure disabled persons more precisely than healthy people. The NEI VFQ-25 was designed to have 12 subscales, but only three (Role difficulties, Near activities and Driving) met the criteria for valid measurement in our study. Bearing in mind that only a small percent of total study population answered driving items we have to be careful in drawing conclusion. Authors who revealed multidimensionality of the NEI VFQ questionnaire by PCA suggested that the NEI VFQ was an instrument with two scales ‘Visual functioning’ and ‘Socioemotional’ [31, 33, 34]. According to this finding we also constructed the visual functioning scale and the socioemotional scale. Our results were similar with the previous reported findings [31, 33]. The psychometric characteristics of the visual functioning scale were slightly better compared to the socioemotional scale. Targeting was suboptimal in both scales. The similar results were found by other authors and indicated that the reengineered versions were not perfect [31, 33, 34]. However, one of the most important tasks in the designing of the questionnaire is to enable that the questionnaire measures only a single underlying construct. This is where the use of Rasch analysis plays a critical role, and has been shown to have higher precision in the evaluation of the quality of the patient-reported outcomes. Bearing in mind that developing of slightly different versions of the same questionnaire can be confusing in some way and may make comparison between studies in different populations difficult, there is a need for valid scales of the English version of the NEI VFQ. Khadka, McAlinden and Pesudovs [38] carried out systematic review of all the available ophthalmic patient-reported outcome (PRO) questionnaires to assess the quality of the following psychometric characteristics: content development, performance of the response scale, dimensionality, measurement precision, validity, reliability, targeting, differential item functioning, and responsiveness. The aim of this review was to inform researchers and clinicians on the choice of the highest quality PRO instrument suitable for their purpose. They recommended six revised scales (Long form visual function scale and Long form socio-emotional scale derived from NEI VFQ-39 and NEI VFQ-25, and Short form visual function scale and Short form socio-emotional scale) and four valid subscales of NEI VFQ (Near vision, Distance vision, Role difficulties and General Health).

Nevertheless, certain limitations of our study may have to be considered. First of all, we used cross-sectional survey to collect data and we were not able to determine long-term change of QoL associated with visual impairment. Second, our study included common ophthalmic diseases and it is unclear whether these findings are applicable to patients with diseases other than cataract, diabetic retinopathy, ARMD and glaucoma. Furthermore, a sample of persons with these ophthalmic conditions may not represent the full clinical spectrum of each disease. Finally, we did not investigate whether the mode of questionnaire administration (e.g. self*-*administered versus face-to-face interview) may influence on the results.

In conclusion, the results of our study indicate that the Serbian version of NEI VFQ-25 is a valid and reliable instrument for the assessment of vision specific QoL in native population according the traditional psychometric methods. However Rasch analysis indicates substantial weaknesses of the questionnaire, particularly in the measurement of dimensionality. Therefore, total score derived from all items seems to be unsuitable and an issue of concern. Measuring of both Visual functioning and Socioemotional constructs should be considered. Despite previous results indicating multidimensionality and some deficiencies in psychometric properties, NEI VFQ-25 is still widely used as an outcome measure among large number of ophthalmologic conditions. This is in some way reasonable because it represents a vision-related quality of life. On the other hand, improving the psychometric properties of the instruments is important and enables researchers to be more precise and accurate in measuring the outcome. Further research should be performed to increase the measurement properties of the the Serbian version of the NEI VFQ-25.

## Additional file

Additional file 1:
**Item analysis.** Number and percentage of missing data and of responses at the floor and ceiling (n = 105) (DOCX 16 kb)

## References

[1] Nutheti R, Shamanna BR, Nirmalan PK, Keeffe JE, Krishnaiah S, Rao GN, Thomas R (2006). Impact of impaired vision and eye disease on quality of life in Andhra Pradesh. Invest Ophthalmol Vis Sci.

[2] Jacobs JM, Hammerman-Rozenberg R, Maaravi Y, Cohen A, Stessman J (2005). The impact of visual impairment on health, function and mortality. Aging Clin Exp Res.

[3] Cahill MT, Banks AD, Stinnett SS, Toth CA (2005). Vision-related quality of life in patients with bilateral severe age-related macular degeneration. Ophthalmology.

[4] Testa MA, Simonson DC (1996). Assesment of quality-of-life outcomes. N Engl J Med.

[5] Tripop S, Pratheepawanit N, Asawaphureekorn S, Anutangkoon W, Inthayung S (2005). Health related quality of life instruments for glaucoma: a comprehensive review. J Med Assoc Thai.

[6] Pesudovs K, Burr JM, Harley C, Elliott DB (2007). The development, assessment, and selection of questionnaires. Optom Vis Sci.

[7] Donovan JL, Brookes ST, Laidlaw DA, Hopper CD, Sparrow JM, Peters TJ (2003). The development and validation of a questionnaire to assess visual symptoms/dysfunction and impact on quality of life in cataract patients: the Visual Symptoms and Quality of life (VSQ) Questionnaire. Ophthalmic Epidemiol.

[8] Massof RW, Rubin GS (2001). Visual function assessment questionnaires. Surv Ophthalmol.

[9] Lundstrom M, Pesudovs K (2011). Questionnaires for measuring cataract surgery outcomes. J Cataract Refract Surg.

[10] Mangione CM, Lee PP, Gutierrez PR, Spritzer K, Berry S, Hays RD (2001). Development of the 25-item National Eye Institute Visual Function Questionnaire. Arch Ophthalmol.

[11] Mangione CM, Berry S, Spritzer K, Janz NK, Klein R, Owsley C, Lee PP (1998). Identifying the content area for the 51-item National Eye Institute Visual Function Questionnaire: results from focus groups with visually impaired persons. Arch Ophthalmol.

[12] Mangione CM, Lee PP, Pitts J, Gutierrez P, Berry S, Hays RD (1998). Psychometric properties of the National Eye Institute Visual Function Questionnaire (NEI-VFQ). NEI-VFQ Field Test Investigators. Arch Ophthalmol.

[13] Rossi GC, Milano G, Tinelli C (2003). The Italian version of the 25-item National Eye Institute Visual Function Questionnaire: translation, validity, and reliability. J Glaucoma.

[14] Nordmann JP, Viala M, Sullivan K, Arnould B, Berdeaux G (2004). Psychometric Validation of the National Eye Institute Visual Function Questionnaire - 25 (NEI VFQ-25) French version: in a population of patients treated for ocular hypertension and glaucoma. Pharmacoeconomics.

[15] Broman AT, Munoz B, West SK, Rodriguez J, Sanchez R, Snyder R, Klein R (2001). Psychometric properties of the 25-item NEI-VFQ in a Hispanic population: Proyecto VER. Invest Ophthalmol Vis Sci.

[16] Toprak AB, Eser E, Guler C, Baser FE, Mayali H (2005). Cross-validation of the Turkish version of the 25-item National Eye Institute Visual Functioning Questionnaire (NEI-VFQ 25). Ophthalmic Epidemiol.

[17] Lin JC, Chie WC (2010). Psychometric validation of the Taiwan Chinese version of the 25-Item National Eye Institute Visual Functioning Questionnaire. J Eval Clin Pract.

[18] Suzukamo Y, Oshika T, Yuzawa M, Tokuda Y, Tomidokoro A, Oki K, Mangione CM, Green J, Fukuhara S (2005). Psychometric properties of the 25-item National Eye Institute Visual Function Questionnaire (NEI VFQ-25). Japanese version. Health Qual Life Outcomes.

[19] Labiris G, Katsanos A, Fanariotis M, Tsirouki T, Pefkianaki M, Chatzoulis D, Tsironi E (2008). Psychometric properties of the Greek version of the NEI-VFQ 25. BMC Ophthalmol.

[20] Simao LM, Lana-Peixoto MA, Araujo CR, Moreira MA, Teixeira AL (2008). The Brazilian version of the 25-Item National Eye Institute Visual Function Questionnaire: translation, reliability and validity. Arq Bras Oftalmol.

[21] Acquadro C, Jambon B, Ellis D, Marquis P, Spilker B (1996). Language and translations issues. Quality of life and pharmacoeconomics in clinical.

[22] ProQuolid patient-reported outcome and quality of life instruments database SF-36 health survey Serbian version. http://www.proqolid.org/. Accessed 20 June 2012.

[23] Chylack LT, Wolfe JK, Singer DM, Leske MC, Bullimore MA, Bailey IL, Friend J, McCarthy D, Wu SY (1993). The Lens Opacities Classification System III. The Longitudinal Study of Cataract Study Group. Arch Ophthalmol.

[24] Grading diabetic retinopathy from stereoscopic color fundus photographs--an extension of the modified Airlie House classification. ETDRS report number 10. Early Treatment Diabetic Retinopathy Study Research Group. Ophthalmol. 1991, 98:786–806. www.ncbi.nlm.nih.gov/pubmed/2062513. 2062513

[25] Cronbach LJ (1951). Coefficient alpha and the internal structure of tests. Psychometrika.

[26] Colton T (1974). Statistics in medicine.

[27] Kramer MS, Feinstein AR (1981). Clinical biostatistics. LIV. The biostatistics of concordance. Clin Pharmacol Ther.

[28] Streiner DL, Norman GR, Streiner DL, Norman GR (1995). Reliability. Health measurement scales : a practical guide to their development and use.

[29] Campbell DT, Fiske DW (1959). Convergent and discriminant validation by the multitrait-multimethod matrix. Psychol Bull.

[30] Andrich D (1978). A rating formulation for ordered response categories. Psychometrika.

[31] Pesudovs K, Gothwal VK, Wright T, Lamoureux EL (2010). Remediating serious flaws in the National Eye Institute Visual Function Questionnaire. J Cataract Refract Surg.

[32] Khadka J, Ryan B, Margrain TH, Court H, Woodhause JM (2010). Development of the 25-item Cardiff Visual Ability Questionnaire for Children (CVAQC). Br J Ophthalmol.

[33] Marella M, Pesudovs K, Keeffe JE, O’Connor PM, Rees G, Lamoureux EL (2010). The psychometric validity of the NEI VFQ-25 for use in a low-vision population. Invest Ophthalmol Vis Sci.

[34] Mollazadegan K, Huang J, Khadka J, Wang Q, Yang F, Gao R, Pesudovs K (2014). Cross-cultural validation of the National Eye Institute Visual Function Questionnaire. J Cataract Refract Surg.

[35] Kielhofner G (2006). Research in occupational therapy : methods of inquiry for enhancing practice.

[36] Pesudovs K, Garamendi E, Keeves JP, Elliott DB (2003). The Activities of Daily Vision Scale for cataract surgery outcomes: re-evaluating validity with Rasch analysis. Invest Ophthalmol Vis Sci.

[37] Lamoureux EL, Pallant JF, Pesudovs K, Hassell JB, Keeffe JE (2006). The Impact of Vision Impairment Questionnaire: an evaluation of its measurement properties using Rasch analysis. Invest Ophthalmol Vis Sci.

[38] Khadka J, McAlinden C, Pesudovs K (2013). Quality assessment of ophtalmic questionnaires: review and recommendation. Optom Vis Sci.

