# Supplementary material for: Validation and cross-cultural adaptation of the National Eye Institute Visual Function Questionnaire (NEI VFQ-25) in Serbian patients
Source: Health Qual Life Outcomes. 2015 Sep 15;13:142. doi: 10.1186/s12955-015-0330-5 (PMC4570616; doi:10.1186/s12955-015-0330-5)
Supplement: Additional file 1: — Item analysis. Number and percentage of missing data and of responses at the floor and ceiling (n = 105) (DOCX 16 kb) [file 12955_2015_330_MOESM1_ESM.docx]

*Additional file 1* Item analysis. Number and percentage of missing data and of responses at the floor and ceiling (n = 105)

| Subscale and item | Missing responses  Number (%) | Floor responses  Number (%) | Ceiling responses  Number (%) |
| --- | --- | --- | --- |
| 1. General health:  5-level health rating | 0 (0.0) | 14 (13.3) | 8 (7.6) |
| 2. General vision:  6-level general vision | 0 (0.0) | 0 (0.0) | 0 (0.0) |
| 3. Mental health:  Amount true : worry | 0 (0.0) | 7 (6.7) | 11 (10.5) |
| 4. Ocular pain:  Amount pain | 0 (0.0) | 0 (0.0) | 55 (52.4) |
| 5. Near vision:  Reading normal newsprint | 0 (0.0) | 15 (14.3) | 7 (6.7) |
| 6. Near vision:  See well up-close | 3 (2.9) | 6 (5.7) | 13 (12.4) |
| 7. Near vision:  Finding objects on crowded  shelf | 0 (0.0) | 2 (1.9) | 33 (31.4) |
| 8. Distance vision:  Reading street signs | 2 (1.9) | 6 (5.7) | 26 (24.8) |
| 9. Distance vision:  Going down stairs at night | 1 (1.0) | 3 (2.9) | 19 (18.1) |
| 10. Peripheral vision:  Seeing objects off to side | 0 (0.0) | 1 (1.0) | 31 (29.5) |
| 11. Social function:  Seeing how people react | 0 (0.0) | 2 (1.9) | 64 (61.0) |
| 12. Color vision:  Difficulty matching clothes | 0 (0.0) | 0 (0.0) | 84 (80.0) |
| 13. Social function:  Visiting others | 7 (6.7) | 2 (1.9) | 61 (58.1) |
| 14. Distance vision:  Going out to movies/plays | 34 (32.4) | 12 (11.4) | 30 (28.6) |
| 15. Driving:  Daylight familiar places | 62 (59.0) | 16 (15.2) | 17 (16.2) |
| 16. Driving:  At night | 77 (73.3) | 3 (2.9) | 4 (3.8) |
| 17. Role limitation:  Accomplish less | 0 (0.0) | 8 (7.6) | 25 (23.8) |
| 18. Role limitation:  Limited in endurance | 0 (0.0) | 2 (1.9) | 27 (25.7) |
| 19. Ocular pain:  Amount time: pain | 0 (0.0) | 0 (0.0) | 67 (63.8) |
| 20. Dependency:  Stay home most of time | 0 (0.0) | 4 (3.8) | 54 (51.4) |
| 21. Mental health:  Amount true: frustrated | 0 (0.0) | 4 (3.8) | 41 (39.0) |
| 22. Mental health:  Amount true: no control | 0 (0.0) | 7 (6.7) | 18 (17.1) |
| 23. Dependency:  Rely too much on other's  words | 0 (0.0) | 1 (1.0) | 53 (50.5) |
| 24. Dependency:  Need much help from others | 0 (0.0) | 1 (1.0) | 61 (58.1) |
| 25. Mental health:  Amount true: embarrassment | 2 (1.9) | 5 (4.8) | 73 (69.5) |
